# OCT-Based Periodontal Inspection Framework

**DOI:** 10.3390/s19245496

**Published:** 2019-12-12

**Authors:** Yu-Chi Lai, Chia-Hsing Chiu, Zhong-Qi Cai, Jin-Yang Lin, Chih-Yuan Yao, Dong-Yuan Lyu, Shyh-Yuan Lee, Kuo-Wei Chen, I-Yu Chen

**Affiliations:** 1 Department of Computer Science and Information Engineering, National Taiwan University of Science and Technology, Taipei 106, Taiwan; cheeryuchi@gmail.com (Y.-C.L.); tbcey74123@gmail.com (C.-H.C.); jimmy.cai3220@gmail.com (Z.-Q.C.); darkgrouptw@gmail.com (J.-Y.L.); chen51202@gmail.com (K.-W.C.); karls820210@gmail.com (I.-Y.C.); 2 Department of Dentistry, National Yang-Ming University, Taipei 112, Taiwan; dongyuan.lyu@gmail.com (D.-Y.L.);; 3 Department of Stomatology, Taipei Veterans General Hospital, Taipei 112, Taiwan; 4 Department of Dentistry, Taipei City Hospital, Taipei 103, Taiwan

**Keywords:** OCT based periodontal inspector, periodontal examination, deep network identifier

## Abstract

Periodontal diagnosis requires discovery of the relations among teeth, gingiva (i.e., gums), and alveolar bones, but alveolar bones are inside gingiva and not visible for inspection. Traditional probe examination causes pain, and X-ray based examination is not suited for frequent inspection. This work develops an automatic non-invasive periodontal inspection framework based on gum penetrative Optical Coherence Tomography (OCT), which can be frequently applied without high radiation. We sum up interference responses of all penetration depths for all shooting directions respectively to form the shooting amplitude projection. Because the reaching interference strength decays exponentially with tissues’ penetration depth, this projection mainly reveals the responses of the top most gingiva or teeth. Since gingiva and teeth have different air-tissue responses, the gumline, revealing itself as an obvious boundary between teeth and gingiva, is the basis line for periodontal inspection. Our system can also automatically identify regions of gingiva, teeth, and alveolar bones from slices of the cross-sectional volume. Although deep networks can successfully and possibly segment noisy maps, reducing the number of manually labeled maps for training is critical for our framework. In order to enhance the effectiveness and efficiency of training and classification, we adjust Snake segmentation to consider neighboring slices in order to locate those regions possibly containing gingiva-teeth and gingiva–alveolar boundaries. Additionally, we also adapt a truncated direct logarithm based on the Snake-segmented region for intensity quantization to emphasize these boundaries for easier identification. Later, the alveolar-gingiva boundary point directly under the gumline is the desired alveolar sample, and we can measure the distance between the gumline and alveolar line for visualization and direct periodontal inspection. At the end, we experimentally verify our choice in intensity quantization and boundary identification against several other algorithms while applying the framework to locate gumline and alveolar line in vivo data successfully.

## 1. Introduction

Periodontal disease occurs frequently in youth and middle aged people [1], and it generally causes gingivitis. Gingivitis results from bacteria or acidic substances eroding gums and alveolar bones, resulting in gum shrinkage, loss of alveolar bone, and tooth root exposure, and finally, it causes periodontitis of fallen teeth. Periodontal disease is hard to diagnose because gums occlude roots and alveolar bones from visual inspection. Therefore, this work aims at developing an automatic non-invasive periodontal inspection framework that can be applied frequently without high radiation and pain.

Traditionally, there are two commonly used periodontal inspection mechanisms, probe based [2,3,4,5] and X-ray based [6,7,8]. First, dentists use a periodontal probe to poke between the gums and the teeth to slip below the gumline in order to reach the junctional epithelium, i.e., the bottom of the periodontal pocket for diagnosis [2,3,4,5]. This is the most commonly used because it is quick and immediate for diagnosis and harmless to the human body. However, dentists totally need to examine six locations for a tooth. When the patient’s teeth are red, swollen, inflamed, and bleeding, the puncture can cause extreme tingling and discomfort. Second, dentists can also examine the distance between the cemento-enamel junction and alveolar bones using X-ray imaging [6,7,8]. This cannot be applied frequently due to the toxic radiation. Therefore, this work adapts non-invasive, gum penetrative, painless, and harmless Optical Coherence Tomography (OCT) for periodontal inspection due to the following benefits. (1) It provides real-time sub-surface imaging at near-microscopic resolution; (2) it requires no preparation of the imaged subjects, and it can image the region of interest without contact or through a transparent window or membrane; (3) it does not emit ionizing radiation. In the past, there was research applying OCT for manually inspecting under-gum dental structures [9,10] and periodontal states [11,12,13]. Mota and Fernandes et al. [14,15,16] applied OCT to characterize the tooth–gingival interface of porcine jaws, teeth of healthy patients, and teeth of patients with periodontal disease by manually processing and labeling the desired periodontal structures. This work automates the identification of the gumline and alveolar line from OCT imaging to provide useful periodontal information for diagnosis.

Finally, we examine the performance of our selected algorithm at each stage against other algorithms. Additionally, we also test our periodontal inspector on an in vivo dataset collected from two subjects for precise detection of the gumline and alveolar line against manually labeled ground truths. Accordingly, we make the following contributions: We design an automatic OCT based periodontal inspection framework, which is non-invasive, harmless, and can be frequently applied. Our system detects the gumline on the amplitude projection along the shooting direction because injected signals decay exponentially with penetration depth to emphasize mostly the characteristics of the top most tissues where the amplitude projection is the accumulation of the interference responses of all penetration depths along a shooting direction for all shooting directions. Additionally, we locate the alveolar line in each slice of the cross-sectional volume. Although deep networks can be directly applied for identification of the gumline and alveolar line, it would require a very large set of scanned data along with a huge amount of the GPU training time. Therefore, we apply Snake segmentation with the extra consideration of neighboring slices to locate regions possibly containing these boundaries in order to have effective training data and reduce the amount of examination. Additionally, we also adapt the truncated direct logarithm of the Snake focused region to transform the scanning volume data to emphasize the regions of interest and their boundaries for easier classification. As demonstrated in the results, our OCT based inspector can properly and efficiently provide useful periodontal conditions to dentists for periodontal disease diagnosis.

## 2. Related Work

This work aims at developing an automatic and frequently applied non-invasive periodontal inspection framework based on OCT. It involves several fields, but due to the length limitation, we restrict our attention only on medical practices in periodontal diagnosis and applications of deep networks to tomography.

Periodontal diagnosis: Xiang et al. [5] indicated that clinically, dentists mainly use three indicators for the diagnosis of periodontal disease. The first is bleeding on probing, i.e., while a dentist pokes the gum with a periodontal probe, it bleeds or does not. The second is pocket depth, which is the distance from the attached gingiva to the junctional epithelium measured with a periodontal probe. However, dentists have a hard time controlling the applied power and angle for precise measurement [2,3,4]. Additionally, it requires six examination locations per tooth. When the patient’s teeth are red, swollen, inflamed, and bleeding, the puncture can cause extreme tingling and discomfort. The final one depends on X-ray imaging to locate and examine the hard alveolar bones [6,7,8]. Although it is non-invasive, X-ray involves high radiation and cannot be applied frequently. Therefore, we develop a gum penetrative OCT scanner with various stages including optical rectification, intensity quantization, tissue identification, and state estimation for periodontal inspection.

Other advanced technologies evaluate whether periodontal treatment is successful based on microbiological testing including fluorescence microscopy [17], flow cytometry [18], the enzyme linked immunosorbent assay [19], and polymerase chain reaction [20]. However, these are very expensive and cannot be reused in the clinic, while our system can be frequently applied to every possible place of every patient. Additionally, the scanned information is directly digitalized for further analysis. Genetic polymorphism [21] uses gene analysis to find potential patients with gingivitis. Other gene analyzing methods use the count of immunoglobulin [22] and interleukin-1 [23] to determine patients susceptible to periodontal disease. However, these results vary with the etiology, growth environment, and other conditions of the constituent bacteria, and thus, they cannot be directly used for gingivitis diagnosis. Our OCT based inspector can directly identify the gumline and alveolar line to give dentists a direct and helpful indication.

There are research efforts focusing on applying OCT imaging to understand the under-gum dental structures [9,10]. Moreover, some groups also take advantage of the gum penetrative abilities of OCT for periodontal inspection [11,12,13]. However, all these methods require manual inspection and examination. Mota et al. [14] examined the periodontal structures of porcine jaws with OCT, while Fernandes et al. [15,16] applied OCT to examine the teeth of patients without/with periodontal disease. However, their analysis required manual image processing and tissue labeling. Although Lai et al. [24] applied OCT to reconstruct the dental surface, they did not aim at periodontal inspection. Our work takes advantages of OCT’s gum penetrative abilities for periodontal inspection without pain while being able to be applied frequently.

Deep tomographic networks on medical images: There are various tomography methods include Magnetic Resonance Imaging (MRI), Computed Tomography (CT), and Optical Coherence Tomography (OCT) targeting various organs. Pereira et al. [25] applied N4ITK [26] to overcome bias field distortion along with imaging statistics for better MRI imaging results and a simple Convolution Neural Network (CNN) to locate possible tumors. Poudel et al. [27] applied a Recurrent Fully Convolutional Neural Network (RFCNN) to identify various hearing components from MRI imaging. Suzuki et al. [28] applied massive training artificial neural networks with voting, and Van et al. [29] combined a neural network and Support Vector Machine (SVM) to identify lung nodules. Fundamentally, these techniques aim at human torsos, having a very limited resolution for teeth, along with their toxic radiation. The imaging processes are totally different to induce different noises, and the image processing and segmentation techniques should be different for good results. There is research [30,31,32] applying OCT to examine retinas. Additionally, Avanaki et al. [33] used networks to estimate the Rayleigh distribution of the scanned data for denoising, and Röhlig [34] used the Multi-scale Convolutional Mixture of Expert (MCME) to locate the regions of interest. However, their target was different from ours. As shown, we develop different rectification, quantization, and segmentation techniques for better results.

## 3. Swept Source Optical Coherent Tomography

**Figure 1:**
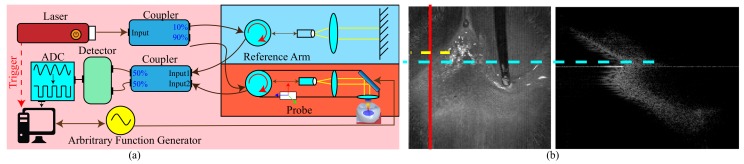
The (**a**) is the top-down ray based interference accumulation for various frequencies, and the (**b**) is the cross-sectional interference for the slice of the scanned volume marked with red. We can determine the alveolar point for each slice marked in cyan from each cross-sectional map, but it is hard to locate the gumline from the same view. However, we find that we can identify the gumline marked in yellow from the amplitude projection.

Optical Coherence Tomography (OCT) is an interferometric and noninvasive 3D volumetric imaging technique [10,30,31,32]. Because it can provide real-time sub-surface imaging without subject preparation and toxic ionizing radiation, it is well suited for studying biological structures. While for imaging, Swept Source Optical Coherence tomography (SSOCT) [35] emits light of various frequencies onto the subject, and the interfered light is collected by the measurement sensor. The ratio of the emitted and received light for various frequencies is used to determine the structural profile of the subject by applying inverse Fourier transform, i.e., a cross-sectional tomograph. This can provide a better depth profile while using less scanning time. Traditionally, this technology is applied for eye examination [32], while Ortman et al. [6] introduced it for alveolar inspection, and Lai et al. [24] used it for tooth scanning and reconstruction. This work uses the hardware described by Lai et al. [24] for gum penetrative inspection of periodontal states as shown in the left of Figure 1.

## 4. Overview

**Figure 2:**
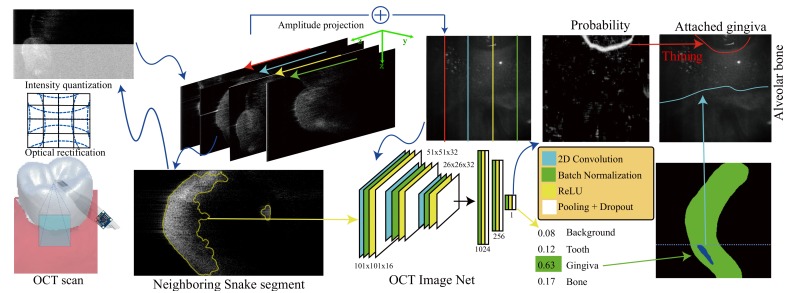
After applying the OCT scanner and signal processing [24], we can gain 3D cross-sectional volumetric interference data. Our system first applies optical rectification and intensity quantization to process the volumetric data. Then, we compute the shooting amplitude projection and apply the OCT net to locate the gumline. Our system uses 2.5D Snake segmentation to locate the Region Of Interest (ROI) of each slice, quantizes it based on the properties of its ROI, and detects the alveolar line using our OCT net. Finally, we analyze the gumline and alveolar line for visualization and diagnosis.

While shooting, we assumed that the applier targeted the probe at the tooth–gingiva boundary and leveled the probe to be perpendicular to gravity. Therefore, as shown in Figure 2, we could define the capturing coordinate based on the scan to have *X* be the direction pointing to the ground, *Y* be the direction to align all slices, and *Z* be the direction in which the OCT shoots, i.e., the shooting direction. Each gum penetrative cross-sectional slice can spatially provide the corresponding response from various depths, and our system intends to segment the responses for identification of gingiva, teeth, and alveolar bones. However, from dentists’ perspective, they generally would like to know the distance in the *Z* direction between the gumline and the alveolar line, as shown in the right of Figure 1. These 3D boundaries are hard to measure. We observed that while OCT emitted at a specific frequency in a specific direction, only tissues at a specific depth could return interference based on the reaching strength, which exponentially decays with the penetrated tissues. Therefore, while accumulating the cross-sectional interferences along a shooting direction for all directions to form an amplitude projection, the top most gingiva or teeth had the strongest interference and showed the dominant effect. As shown in the left of Figure 1, because gingiva and teeth have different hardness for obviously different air–gingiva and air–teeth responses, there exists an obvious boundary between teeth and gingiva, i.e., the gumline. s for obviously different air–gingiva and air–teeth responses, there exists an obvious boundary between teeth and gingiva, i.e., the gumline. As a result, this work intends to locate the gumline from the maximal projection and use it as the basis line for periodontal inspection while using the nine-axis sensor to locate the slicing direction in order to locate the corresponding alveolar line for better periodontal inspection.

Figure 2 illustrates our entire inspection process. After applying the OCT scanner [24] to gain the spatial interference patterns of various frequencies, we could use fast Fourier transform to reconstruct a 3D cross-sectional volume for the target region. Our system applied the hybrid optical rectification [24] of traditional camera calibration and Thin-Plate Spline (TPS) to correct the lens distortions. Since newly available deep networks [36,37,38] proved their ability to identify various regions from noisy images, our system adapted a deep network, the OCT net, to locate the gumline automatically, i.e., the gingiva–tooth boundary, and alveolar line, i.e., the gingiva–alveolar boundary, from the amplitude projection and slices of the scanning volume, respectively. While having enough training data, deep networks should be able to take the interference variations of personal differences, noises, scanning distances, and other factors into consideration. However, it is hard to collect a very large number of scanning volumes because of the huge amount of man power for labeling and fulfilling the laws of clinical trials. Therefore, we applied the truncated direct logarithm [39] to have the interference values in [0,255] for identification of the gumline and Region Of Interest (ROI). Then, our system accumulated the interference responses along the capturing *Z* direction, i.e., of various depths, to form the amplitude projection. Our OCT image network identified gingiva from the projection for the gumline. Generally, air lying on the top of each slice provides very small interference, and the teeth roots located in the bottom half also provide little responses because of exponential decaying. Both provide little information to the deep network, and thus, we adapted Snake segmentation [40] to locate informative regions for better quantization, sample collection, and effective classification. Our system applied the truncated direct logarithm [39] according to the properties of the Snake focused region in order to emphasize boundaries. We sliced the scanning volume along the *X* direction of the capturing coordinate and used another OCT image network to identify gingiva, teeth, and alveolar bones for the alveolar line. Finally, our system aligned the detected gumline and alveolar line for analysis and visualization along the *X* direction of the capturing coordinate for diagnosis.

## 5. Algorithmic Details

Although OCT imaging can penetrate gums, its captured data are generally noisy. In order to estimate periodontal states precisely, we must optically rectify and quantize the captured data while applying deep networks for precise boundary identification. Finally, we analyzed and visualized the detection lines for periodontal diagnosis. The following details these stages.

### 5.1. Optical Rectification

Infrared rays were emitted and received through the lens for possible induction of optical distortions. Therefore, we followed the same hybrid calibration process of traditional camera calibration and Thin-Plate Spline (TPS) [24] for the OCT-to-world transformation. We first set a given set of *N* sampling locations, $\left\{\cdots ,\left(S_{i},T_{i},X_{i},Y_{i}\right),\cdots \right\}$ where (S,T) denotes the OCT captured coordinate and (X,Y) denotes the stage coordinate. We first determined $\left(k_{1},k_{2},k_{3},p_{1},P_{2}\right)$ by solving $\left[\begin{array}{c} x\\ y \end{array}\right]=\left(1+k_{1}r^{2}+k_{2}+k_{3}r^{4}\right)\left[\begin{array}{c} s\\ t \end{array}\right]$ $\left[\begin{array}{c} x\\ y \end{array}\right]=\left[\begin{array}{c} s+2p_{1}t+p_{2}\left(r^{2}+2s^{2}\right)\\ t+2p_{1}\left(r^{2}+2t^{2}\right)+2p_{2}s \end{array}\right]$, where $r=\sqrt{s^{2}+t^{2}}$, for correcting radial and tangential distortions. Then, we formed two as-harmonic-as-possible functions, X(U,V) and Y(U,V), based on *N* sampling locations, $\left\{\cdots ,\left(U_{i},V_{i},X_{i},Y_{i}\right),\cdots \right\}$ where (U,V) denotes the corrected coordinate and (X,Y) denotes the stage coordinate. Our system minimized the bending energy of Thin-Plate Spline (TPS) as $\int \int_{\Omega }f_{UU}^{2}+2f_{UV}^{2}+f_{VV}^{2}dUdV$, where *f* is for *X* and *Y*, respectively. We could then utilize two functions to estimate its true world coordinate.

### 5.2. Locate Effective Regions with 2.5D Snake

The resolution of each slice was 250×1024, and while putting them into training and testing, we had the following three issues. First, the distance of the scanner to the target region varied, inducing variations in the slice; this in turn generally required more training data for more precise prediction. Second, the magnitude of the cross-section responses varied depending on the cross-section information along the *Z* direction of the capturing coordinate. Although deep networks can automatically find the best relationship among various pixels and cross-sections, but would require a large amount of marked data, which is generally time consuming and hard to achieve. Third, although the entire slice had more examples, those portions of air and the bottom tooth generally had very little responses, i.e., these parts induced too many background examples to bias the training. Therefore, we first calibrated and quantized the slice to emphasize the boundaries for easy recognition. Additionally, we adapted Snake segmentation [40] to locate those regions of interest whose interferences were far from zero in order to remove too many background training examples. This section first gives the details of our adapted Snake segmentation, and the next section details the intensity calibration and quantization. Snake segmentation [40] can locally find the cut to separate two materials, while GrabCut [41] must globally solve the optimal graph, which is more time consuming and hard to parallelize. Thus, we used a flexible 2D curve, C, moved inside a slice to minimize the designed energy for the depth response image in order to locate the boundary points. The energy is as $E=E^{int}+E^{data}+E^{neighbor}$ where $E^{int}=C^{\prime 2}+C^{\prime \prime 2}$ is the internal energy based on the continuity and curvature of the Snake curve, $E^{data}$ is the data energy directly using the depth response for the indication of another material, and $E^{neighbor}$ is the neighboring energy to take the boundary of the previous slice into consideration. We express the data term as $E^{data}=w^{intensity}E^{intensity}+w^{edge}E^{edge}+w^{direction}E^{direction}$ where $w^{intensity}$, $w^{edge}$, and $w^{direction}$ are weights for each term, where we set them as 0.3, 0.3, and 0.4, $E^{intensity}$ is the intensity energy term based on the average value of a 3×3 box kernel, $E^{edge}$ is the edge energy term based on its gradient of a 3×3 Gaussian kernel, $E^{direction}=\frac{\partial \theta }{\partial {\vec{n}}^{⊥}}$ is the direction energy, where θ is the gradient direction, and ${\vec{n}}^{⊥}$ is the normal of the boundary, to indicate the deviation between the gradient and the boundary normal because they should be perpendicular to each other when converging. While training and segmenting, we used slices of the 3D volumetric interference map. However, it was actually a 3D volume, and neighboring slices should have spatial coherence. If we did not take this into consideration, the system could easily get stuck at local minima, containing too much undesired background. Since the boundary surfaces should be smooth locally, the boundary of two slices should be similar. In other words, the distance of the current boundary point to the boundary of the previous slice should be minimized. Thus, we have the neighbor energy as $E^{neighbor}=\frac{1}{D(i,j)}$ where D(i,j) is the distance to the neighboring boundary. In each slice, we had the maximal ratio boundary proposed by Lai et al. [24] as the initial curve C and advanced it sequentially until converging.

### 5.3. Inference Intensity Calibration and Quantization

The injected energy of our probe spatially varied with the injected directions, but it did not vary temporally. Therefore, we first used the OCT scanner to capture a plane platform, computed the amplitude projection, and used the projection to calibrate the injected energy to ensure response consistency across pixels. Based on interviews with analysts for OCT imaging, while quantizing the slices, the results, which reach the following criteria, can make them more easily locate teeth, gingiva, and alveolar bones. First, the left and right of each slice consisted of air and teeth, respectively, and its interference response should be very small. Second, it is important to identify the gumline and alveolar line, and thus, the gradient across the boundaries should be high for easy identification. Finally, while penetration depth increases, the interference response decays, i.e., responses inside the homogeneous material should be similar. Here, the goal of quantization, mapping real values to a series of fixed gray levels, is for data visualization. Generally, there are four commonly used methods including equal interval (linear mapping), equal probability (histogram equalization), minimum variance, and histogram hyperbolization [39]. We adapted truncation logarithm quantization, which takes both dynamic range determination and noise reduction into consideration to select a proper section in the responses and transform it to the visible range for later deep network training and identification. Additionally, while quantizing the volume, we had three different choices based on the quantized size: pixel based, slice based, and volume based. Pixel based quantization only considering a single pixel may lose spatial coherence, and the volume based one taking the entire data into consideration may miss consideration of local details. Additionally, our system applied our OCT net on 2D slices, and thus, it was more important to make the characteristics of each slice distinct. We quantized the data based on slice information by first computing the logarithm of all pixels in the scanning volume. Next, we applied the adapted 2.5D Snake to locate the ROI of each slice. For each slice, we established its histogram in the located ROI, found the low mode of the distribution, fit the mode with a normal distribution for the mean and standard deviation, used the mean as the truncated threshold, $T^{q}$, and set the maximal logarithmic intensity, $M^{q}$, as the maximum value of the ROI. For those smaller than $T^{quantization}$, we set their values as zero; for those larger than $M^{q}$, we set their values as 255; otherwise, we linearly mapped them into [0,255].

### 5.4. Top Down Gingival Boundary Identification

**Figure 3:**
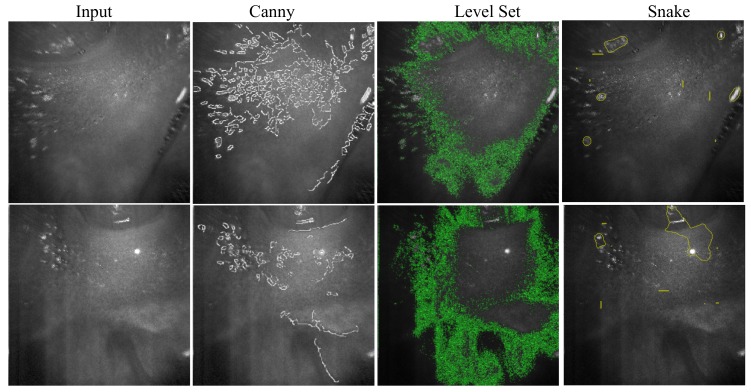
This shows the boundary of gingiva and teeth, i.e., the gumline, identified by canny [42], LevelSet [43], and Snake [40].

As discussed in Section 4, the gumline, an important periodontal evaluation criterion, reveals itself as an obvious boundary of the gingiva and teeth in the amplitude projection. While using traditional segmentation techniques including canny [42], LevelSet [43], and Snake [40], as shown in Figure 3, the results were unsatisfactory due to their noisy nature. Therefore, we decided to apply the newly available deep learning methods for its identification. Generally, SegNet [38] should be able to accomplish this task, but it requires a large amount of data for training where manually labeling data is time consuming and in vivo data collection on patients requires strenuous and cumbersome official application to the government administration. Therefore, this work first collected the data from our teammates, and a professional analyst manually labeled the gingiva and teeth. Additionally, instead of using the entire map of 250×250 for training, we applied the sliding window mechanism of a window size of 101×101 to slide through the collected maps for a reduction of the parameter number and increasing the number of data where 101 was chosen based on our test on various sizes using our collected data. As shown in Figure 2, our network first adapted the encoder structure of SegNet [38] with three stages for extracting important features and added three fully connected convolutional stages for classification. The encoder retained higher resolution features while reducing the number of parameters for a smaller training set, and the extracted features were fed into the fully connected decision network for integrated classification. Each encoder stage convolved the data with a filter bank to have feature maps and batch normalized them. Then, it applied rectified linear non-linearity (ReLU), $max(0,\bar{x})$, on each element and max pooling of a 2×2 window and a stride of 2 for sub-sampling of a factor of two. These two steps aimed at translation invariance over small spatial shifts and encoding larger image context. Additionally, we added in a random drop-out step for better efficiency and accuracy. We had three stages for robust classification. The output of the encoder was linearized for classification with ReLU, max pooling, and random drop-out. At the end, the decision stage output the probability of the classification. This work used Mean Squared Error (MSE) as the loss function and the Adam gradient optimizer [44] for training optimization. While directly plugging the data into training, the learning bias toward background became too large. Therefore, we first separated the data into two categories, gingiva and background. For each iteration, our system randomly and evenly selected 128 examples from both categories for training due to the limitation of the GPU memory. The process repeated until it converged.

#### Thinning for the Gingival Boundary

After applying our OCT network on the amplitude projection, we had a probability distribution of the gingiva. Directly using a threshold easily results in disconnected, thick boundaries. Therefore, we applied the thinning method proposed by Zhang et al. [45] in the following steps. First, we binarized the probability map, P, with a threshold, $T^{thin}$, to get B where $T^{thin}=0.50$ in our experiment. Second, we went through all pixels to set the value of a pixel, (x,y), to zero if the following conditions were satisfied:

(1) $$\begin{array}{l} \delta^{min}<=\sum_{i=1}^{8}B\left(P_{i}\right)<=\delta^{max}\\ \sum_{i=2}^{7}A\left(P_{i},P_{i+1}\right)=1,\;A\left(p_{i},p_{i+1}\right)=\left\{\begin{array}{ll} 1 & \mathrm{if}\;p_{i}=0\;\mathrm{and}\;p_{i+1}=1\\ 0 & \mathrm{otherwise} \end{array}\right.\\ B\left(P_{1}\right)B\left(P_{3}\right)B\left(P_{5}\right)=0\\ B\left(P_{3}\right)B\left(P_{5}\right)B\left(P_{7}\right)=0 \end{array}$$

where $\delta^{min}$ and $\delta^{max}$ are two user specified parameters and set to be two and six and $P_{i}$ are the neighbors of any given pixel (x,y), starting from the top neighbor and ordering clockwisely. Finally, we repeated the second step until the result remained the same. The thinning process continued until the result remained the same in one iteration.

### 5.5. Volumetric Alveolar Bone Boundary Detection

Our system identified the alveolar line by locating the alveolar bones, which reveal themselves as brighter spots in the slices using the same OCT net. The slice data contained a large portion of background, air, and teeth, and while directly plugging into the state-of-the-art SegNet [38], the net intended to label each pixel as background to have low loss. This required repeatedly adjusting the parameters for better results, and it was time consuming for each iteration. Thus, we reduced the training bias by using 2.5D Snake to locate the ROI as discussed in Section 5.2. Then, our system applied the sliding window mechanism for segmentation by finding its bounding box and zero padding the boundaries to have all pixels as training examples to create a set of images with a label of background, teeth, gingiva, or alveolar bones. In order to have an even number for each category, we first determined the number based on the allowed memory. Then, we then randomly and evenly selected 64 training examples from four categories respectively for training in order to avoid bias for each iteration. The process repeated until converging. While classifying, we zero padded the bounding box of ROI to ensure that every interesting pixel could be classified.

## 6. Results

Our OCT based periodontal inspector could non-invasively examine the periodontal conditions. We first used the OCT scanner to collect a set of tooth scans targeting the gumline of subjects. Then, we designed ablation studies to evaluate our chosen stages. Finally, we also analyzed its prediction precision on the detected gumlines and alveolar lines against ground truths. All the results in this section were run under a computer with a CPU with Intel Xeon E5-2698 v4 2.2 GHz (20 cores), 256 G DD4 memory, and 4 NVidia Tesla V100.

### 6.1. Periodontal Dataset

**Figure 4:**
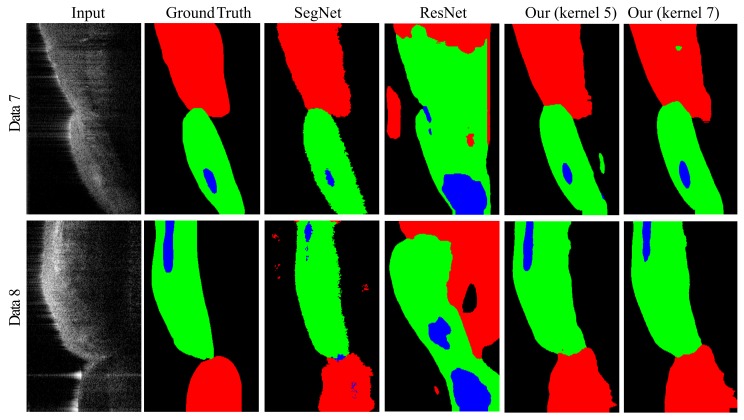
This shows the volumetric segmentation results. From left to right are the inputs, ground truths (marked manually), and the results of SegNet [38], ResNet [37], ours with a kernel size of give, and ours with a kernel size of seven. From top to bottom are the central slices from Validating Data 7 and 8.

We collected 18 OCT in vivo scans, whose resolution was 250×250×1024, from two subjects, whose ages were 23 and 40, respectively, and whose gums were healthy, targeting the tooth–gingiva boundaries. We selected nine random sites from each subject. An analyst manually went through these 18 amplitude projections of a resolution of 250×250 to label the gumlines and regions of gingiva and teeth. Generally, it took about 15 s for an analyst to quantize the scan, 30 s to label the gumline on the projection, and 184 s to label gingiva and tooth regions on a slice. In other words, it took 46,000 s for labeling a whole scan. Then, for each slice of a resolution of 250×1024, the analyst also labeled the regions of the background, gingiva, alveolar bones, and teeth, as shown in Figure 4. Later, our system adapted the sliding window mechanism to have a larger dataset by zero padding the boundaries to use all pixels of the 2D amplitude projection and 3D slices fully. To train, test, and validate the deep network, we randomly chose 60% for training, 30% for testing, and 10% for validation. While actually performing for identification of the gumline and alveolar line, we zero padded the map to extend its width and height to have an output of the same dimension as the input.

### 6.2. Ablation Study in Locating Regions of Interest

**Figure 5:**
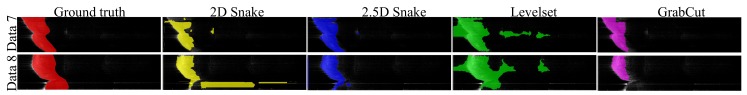
From left to right are manually labeling (red), traditional Snake [40] (yellow), ours (blue), LevelSet [43] (green), and GrabCut [41] (pink).

Our framework proposed 2.5D Snake segmentation to locate regions of interest for removal of redundant background regions. In order to evaluate its effectiveness, we conducted a comparison against the commonly used 2D Snake [40], LevelSet [43], and GrabCut [41]. We adapted 2D Snake [40], LevelSet [43], and GrabCut [41] from the OpenCV library to our framework and used their default settings to locate effective regions on each slice, as shown in Figure 5. On average, traditional Snake [40] took 0.205 s, 2.5D Snake 0.212 s, LevelSet [43]22.9 s, and GrabCut [41]0.486 s. We took the analyst labeled data and computed the Intersection over Union (IoU) between the ground truth and various location methods, as shown in Table 1, where $IoU(A^{D},A^{GT})=\frac{A^{D}\cap A^{GT}}{A^{D}\cup A^{GT}}$, $A^{D}$ is the area of detection, and $A^{GT}$ is the area of ground truth. Generally, 2D Snake [40], LevelSet [43], and GrabCut [41] only start from the same initial condition and consider the properties per slice. These make them easily stuck in noisy regions, and they take longer to converge. Our algorithm used the detected boundary of neighboring slices as an optimal term. This can help Snake to walk over those disturbances for better results while comparing to traditional Snake segmentation. While comparing to LevelSet, our algorithm was simpler, faster, and more stable. The adapted Snake was simpler and had better locating rates than GrabCut.

### 6.3. Ablation Study in Intensity Quantization

In order to reduce the required training data size, we normalized the interference slices for easier classification. We would like to evaluate its effectiveness, and thus, we first designed an evaluation metric based on the criteria described in Section 5.3 as follows.

(2) $$S^{quan}=w^{BG}E^{BG}+w^{BO}E^{BO}+w^{T}E^{T}$$

where $E^{BG}$, $E^{BO}$, and $E^{T}$ are the evaluation metrics for the background, boundary, and target criteria, and $w^{BG}$, $w^{BO}$, and $w^{T}$ are their corresponding weights. This work set $w^{BG}=1.0$, $w^{BO}=0.1$, and $w^{T}=0.1$ for our experiment because the contrast across the boundary had a major influence on identification. First, Lai et al. [24] provided a boundary detection mechanism by locally connecting the first local gradient maximum in the *z*-direction. After penetrating any tissue, the signal decayed exponentially, and therefore, we used a threshold, $T^{quan}$, to locate the other sized region of interest boundary. Different quantization algorithms may result in different brightness distributions, and thus, we computed the histogram of the preprocessed slice and set $T^{quan}$ to be the third quartile of the first mode. We related the IoU of the located background to the ground truth, $IoU^{BG}$, to the background term as $E^{BG}=1-IoU^{BG}$. Our system used the sum of all *Z*-direction distance to the boundary band as the boundary term as $E^{BO}=\sum_{N^{slices}}\sum_{N^{Y}}D^{B}$ where $D^{B}$ is the *Z*-direction distance between that detected and the ground truth. $E^{T}$ basically indicated the approximation to the exponential decays inside the tissues between that quantified and ground truth. This can be approximated by the brightness distribution, and thus, we computed cumulated histograms inside the detected and labeled target regions, computed their correlation, and set the deviation to one as $E^{T}$. We compared our adapted truncated direct logarithm against three commonly used quantization algorithms including c-means minimum distortion [46] to maximize the variance, information expansion [39] to equalize the histogram, and maximum entropy [39] to minimize the information loss. On average, the truncated direct logarithm took 0.0614 s, minimum distortion 58.4 s, information expansion 0.122 s, and maximum entropy 0.850 s. The results are shown in the right of Table 1. Generally, our selected algorithm was simpler and more efficient while its performance was generally more stable and robust to preserve the major boundaries and important tissue regions.

### 6.4. Periodontal Inspection

**Figure 6:**
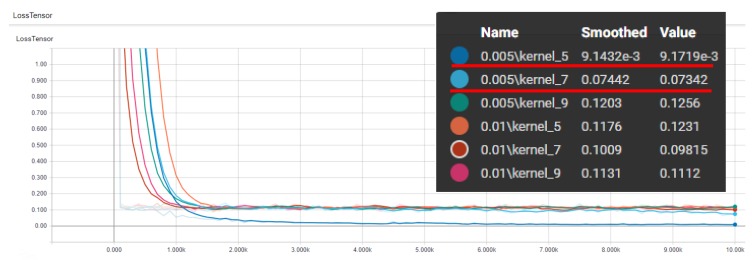
This shows the loss curve of the learning process for our OCT image network while using the combination of two learning rates, $1\times 10^{-2}$ and $5\times 10^{-3}$, and three kernel sizes, 5, 7, and 9.

Our OCT network had several important parameters including the learning rate and the kernel size. While observing past deep research, we found that the learning rate was generally selected between $1\times 10^{-2}$ and $5\times 10^{-3}$ and the kernel size was generally selected among 5, 7, and 9. Therefore, we tested various combinations of these parameters for our network, as shown in Figure 6. Generally, the network converged roughly between 1000 and 2000 iterations, and it could perform better while having a learning rate of $5\times 10^{-3}$ and its kernel size of five and seven. Therefore, we used these parameters for identification in both 2D projection and 3D slices.

**Figure 7:**
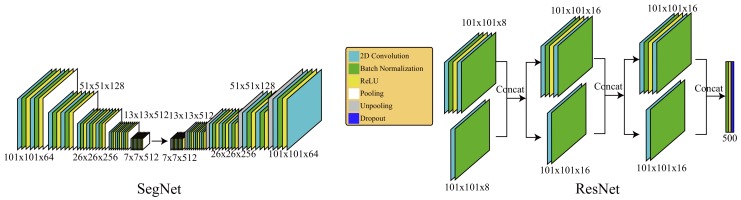
This shows the adapted structures of SegNet [38] and ResNet [37] in this study.

In order to understand the effectiveness of our OCT network, we conducted a comparison against two commonly used networks, SegNet [38] and ResNet [37]. Using 12 accumulation maps to train, 3 to test, and 3 to validate, SegNet [38] did not have enough information for good segmentation. Therefore, we used the same sliding set with a resolution of 101×101. According to the resolution, we reduced the number of layers in the encoder and decoder of SegNet as shown in the left of Figure 7. While labeling gingiva, we zero padded the amplitude projection to 303×303, cut the padded results into 3×3 tiles with a resolution of 101×101, applied the trained SegNet on each of them, and stitched them into the final result. We also reduced the number of layers and adjusted the structure of ResNet [37], as shown in the right of Figure 7 according to the resolution of 101×101, and trained the net with the same set. Then, our system zero padded the projection to 350×350 and applied the trained ResNet to each valid sliding region of 101×101 for gingival classification.

Similarly, we chose to have the same training set for the 3D volumetric slices for SegNet [38] and ResNet [37]. While using SegNet to label teeth, gingiva, alveolar bones, and background, we zero padded the interesting axis aligned bounding box of the region of interest detected by the adapted Snake to have tiles with a resolution of 101×101, applied the trained SegNet on each of them, and stitched them into the final result. Similarly, our system zero padded the bounding box to ensure the classification of all interesting pixels and applied the trained ResNet to each valid sliding region of 101×101 for classification. Figure 4 shows the segmentation results of SegNet [38], ResNet [37], Ours with a kernel size of five (Ours-5), and Ours with a kernel size of seven (Ours-7). We also computed the average IoU of SegNet [38], ResNet [37], and ours with kernel sizes of five and seven, as shown in Table 2. After training, SegNet could perform well on the testing datasets, but while applying it to the validating datasets, its performance deteriorated quickly. This may be due to the noisy nature of the OCT data. ResNet [37] performed comparatively well as our OCT net in 2D amplitude projections, but our net outperformed ResNet in the 3D slices. Generally, our selected resolution was not large enough to demonstrate its strength. In contrast, our simplified OCT network could perform better by selecting important features from each patch and determining its labeling by integrating these correlated features.

**Figure 8:**
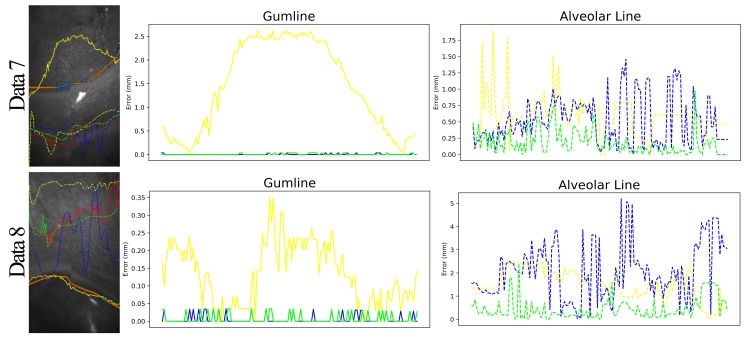
The left shows the gumline in solid lines and the alveolar line in dotted lines of Data 7 (the top) and 8 (the bottom) detected by an analyst in red, SegNet [38] in yellow, ResNet [37] in green, and ours in blue. The middle and right show the deviation analysis against the manually labeled ones for the gumline and the alveolar line, respectively.

Since we intended to have a non-invasive inspection of the periodontal states, our system could directly draw the detected gumline and alveolar line on the top down accumulation maps as shown in Figure 8. At the same time, we also show the detection results of SegNet [38],and ResNet [37] along with manually marking. On average, our system took 1.62 s for scanning, 0.123 s for rectification, 0.0166 s for normalization and quantization, 0.212 s for ROI location, 6.12 s for gumline detection, and 4.85 s for segmentation in each slice. From scanning to visualization, it would take about 20 min while using a general computer. While using the NVidia DGX station, the process could accelerate to 2 min. Clinically, dentists care more about the measurement in the gravity direction, and therefore, we computed the distance between the detected boundaries of SegNet [38], ResNet [37], and ours in the gravity direction to the manually marked ones for precise analysis. Table 3 shows the mean and maximal deviations of SegNet [38], ResNet [37], and ours.

## 7. Conclusions

This work proposed a non-invasive framework for frequent periodontal inspection by estimating the gumline and alveolar line of the target region using optical coherence tomography. Our system optically rectified the scanning results for precise measurement. Furthermore, our system introduced newly available deep networks for boundary identification while using Snake segmentation and intensity calibration and quantization to locate possible boundary regions and signal ranges in order to reduce the required amount of training data and enhance the training efficiency. The results showed that our system could provide reliable estimation of both lines while compared to manually labeled results. However, the proposed system was not without limitations. There are a few future research directions. First, currently, our deep networks works on 2D images for both amplitude projections and 3D interference slices. However, the scanning volumes were actually 3D data, and we would like to apply 3D deep networks in order to take neighboring slices into consideration for possibly better segmentation accuracy. Second, the cemento-enamel junction is the bottom of the periodontal pocket, and dentists locate it by splitting suspended gingiva with a probe. However, the suspended gingiva generally are attached to the teeth, and this cannot be identified by OCT, currently. In other words, our system currently still cannot automatically identify the bottom of the periodontal pocket, i.e., junctional epithelium, in order to estimate the pocket depth because dentists cannot provide the proper indication for detection. Thus, we would like to follow the protocols used in the manual inspections [14,15,16] to locate the bottom in the OCT scans using the examining probe robustly. Later, we can use these marked OCT scans to have a better understanding in order to find good criteria for its identification. Third, while using the nearest alveolar point from the gumline point at each slice is suboptimal, we should be able to improve the precision by reconstructing the alveolar bones and searching for the optimal alveolar line on the surface according to the gumline. Fourth, currently, we have only collected samples from two healthy individuals. In order to evaluate the effectiveness, we would like to get the governmental approval to apply this on patients and collect various samples from various individuals. Fifth, because we designed our inspection framework into various stages, we only need to modify the interference quantization stage for acquiring data while a commercial OCT system should provide the rectification and interference calibration. After quantization, theoretically, the following stages should have similar performance. We would like to seek a commercial probe to examine the effectiveness of our system. However, if the characteristics do not match the requirement of our net, appliers would be required to collect enough scans and label the projection and all slices of these scans.

## Figures and Tables

**Table 1:** The left half of the table shows the slice based IoU for 2.5D Snake, 2D Snake [40], LevelSet [43], and GrabCut [41]. The right half shows the average penalty score for our adapted Truncated Direct Logarithm (TDL), c-means Minimum Distortion [46] (MD), Information Expansion [39] (IE), and Maximum Entropy [39] (ME).

	ROI				Quantization			
	2D Snake	2.5D Snake	LevelSet	GrabCut	TDL	MD	IE	ME
Data 1	0.492	0.703	0.669	0.564	4.914	5.887	5.895	18.36
Data 3	0.505	0.605	0.356	0.574	14.86	14.02	13.76	12.90
Data 7	0.560	0.760	0.519	0.656	10.06	13.41	13.19	15.65
Data 8	0.393	0.489	0.546	0.353	15.24	18.75	19.10	19.15

**Table 2:** This shows the average IoU of Data 1 and 3 for testing and Data 7 and 8 for validating while using SegNet [38], ResNet [37], Ours with a kernel size of 5 (Ours-5) where we only tested it on 3D slices, and Ours with a kernel size of 7 (Ours-7).

	SegNet		ResNet		Ours-7		Ours-5	
	2D	3D	2D	3D	2D	3D	2D	3D
Data 1	0.960	0.489	0.971	0.743	-	0.729	0.977	0.770
Data 3	0.944	0.582	0.982	0.164	-	0.507	0.979	0.623
Data 7	0.384	0.324	0.987	0.0467	-	0.171	0.987	0.411
Data 8	0.287	0.270	0.973	0.380	-	0.575	0.964	0.678

**Table 3:** This shows the MSE of the detected gumline and alveolar line of Data 1 and 3 for testing and Data 7 and 8 while using SegNet [38], ResNet [37], and ours with a kernel size of 7 against the ground truths in the units of mm where Gin. denotes the gumline and Alv. denotes the Alveolar line.

	SegNet				ResNet				Ours			
	Gin.		Alv.		Gin.		Alv.		Gin.		Alv.	
	Mean	Max	Mean	Max	Mean	Max	Mean	Max	Mean	Max	Mean	Max
Data 1	$3.23\times 10^{-1}$	1.68	1.93	5.82	$1.00\times 10^{-2}$	$6.17\times 10^{-2}$	$1.57\times 10^{-1}$	$6.93\times 10^{-1}$	$5.19\times 10^{-3}$	$3.41\times 10^{-2}$	$2.23\times 10^{-1}$	1.69
Data 3	$6.14\times 10^{-2}$	$3.69\times 10^{-1}$	$3.99\times 10^{-1}$	$9.67\times 10^{-1}$	$3.59\times 10^{-3}$	$3.03\times 10^{-2}$	2.62	5.14	$4.47\times 10^{-3}$	$5.47\times 10^{-2}$	$6.25\times 10^{-1}$	$9.52\times 10^{-1}$
Data 7	1.27	2.62	$4.52\times 10^{-1}$	1.89	$7.45\times 10^{-3}$	$3.76\times 10^{-2}$	$4.43\times 10^{-1}$	1.45	$5.82\times 10^{-3}$	$3.76\times 10^{-2}$	$2.17\times 10^{-1}$	$9.76\times 10^{-1}$
Data 8	$1.80\times 10^{-1}$	$5.22\times 10^{-1}$	1.25	2.52	$6.40\times 10^{-3}$	$3.56\times 10^{-2}$	2.14	5.19	$8.20\times 10^{-3}$	$5.21\times 10^{-2}$	$4.62\times 10^{-1}$	2.40

## Abbreviations

The following abbreviations are used in this manuscript:

OCT	Optical Coherence Tomography
GPU	Graphics Processing Unit
4PCS	Super Four Point Congruent Set
FFT	Fast Fourier Transform
CT	Computed Tomography
MRI	Magnetic Resonance Imaging
SSOCT	Swept Source Optical Coherent Tomography
AFG	Arbitrary Function Generator
ADC	Analog-to-Digital Converter
TPS	Thin-Plate Spline
RANSAC	RANdom SAmple Consensus
DT	Transfer a slice of scanning data (DT)
Re	optical Rectification (Re)
BD	Boundary Detection
